# Publisher Correction: Toxoplasma effector TgROP1 establishes membrane contact sites with the endoplasmic reticulum during infection

**DOI:** 10.1038/s41564-025-02256-5

**Published:** 2025-12-22

**Authors:** Chahat Mehra, Jesús Alvarado Valverde, Ana Margarida Nogueira Matias, Francesca Torelli, Tânia Catarina Medeiros, Julian Straub, James D. Asaki, Peter J. Bradley, Katja Luck, Steffen Lawo, Moritz Treeck, Lena Pernas

**Affiliations:** 1 Metabolism of Infection Group, Max Planck Institute for Biology of Ageing, Cologne, Germany; 2 Institute of Molecular Biology (IMB) gGmbH, Mainz, Germany; 3 Cell Biology of Host-Pathogen Interaction Laboratory, Gulbenkian Institute for Molecular Medicine, Lisbon, Portugal; 4 The Francis Crick Institute, London, UK; 5 Department of Microbiology, Immunology & Molecular Genetics, University of California, Los Angeles, CA USA; 6 Molecular Biology Institute, University of California Los Angeles, Los Angeles, CA USA; 7 Max Planck Institute for Biology of Ageing, Cologne, Germany; 8 Howard Hughes Medical Institute, University of California Los Angeles, Los Angeles, CA USA

**Keywords:** Endoplasmic reticulum, Parasite host response, Mitochondria, Cellular microbiology

Correction to: *Nature Microbiology* 10.1038/s41564-025-02193-3, published online 25 November 2025.

In the version of the article initially published, the fourth paragraph of the Discussion (beginning “Are other effectors recruited to host ER-*Toxoplasma* MCS?”) and the reference “Romano, J. D. et al. *Toxoplasma gondii* VIP1 mediates parasitophorous vacuole–host endoplasmic reticulum interactions to facilitate parasite development. *Nat. Microbiol*. 10, 3315–3330 (2025)” were missing and have now been added to the HTML and PDF versions of the article.

