# Supplementary material for: Toxoplasma effector TgROP1 establishes membrane contact sites with the endoplasmic reticulum during infection
Source: Nat Microbiol. 2025 Nov 25;10(12):3331–45. doi: 10.1038/s41564-025-02193-3 (PMC12669048; doi:10.1038/s41564-025-02193-3)
Supplement: Supplementary file 1 — Supplementary Figures 1–5. [file 41564_2025_2193_MOESM1_ESM.pdf]

nature microbiology

Supplementary information https://doi.org/10.1038/s41564-025-02193-3

Toxoplasma effector TgROP1 establishes membrane contact sites with the endoplasmic reticulum during infection

In the format provided by the authors and unedited

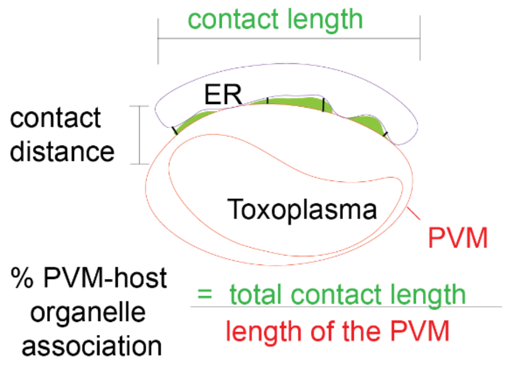

Supplementary Fig. 1: Schematic of host ER-*Toxoplasma* MCS analysis

Schematic of analyses of membrane contact sites (MCS) between host ER and *Toxoplasma* parasite vacuole membrane (PVM).

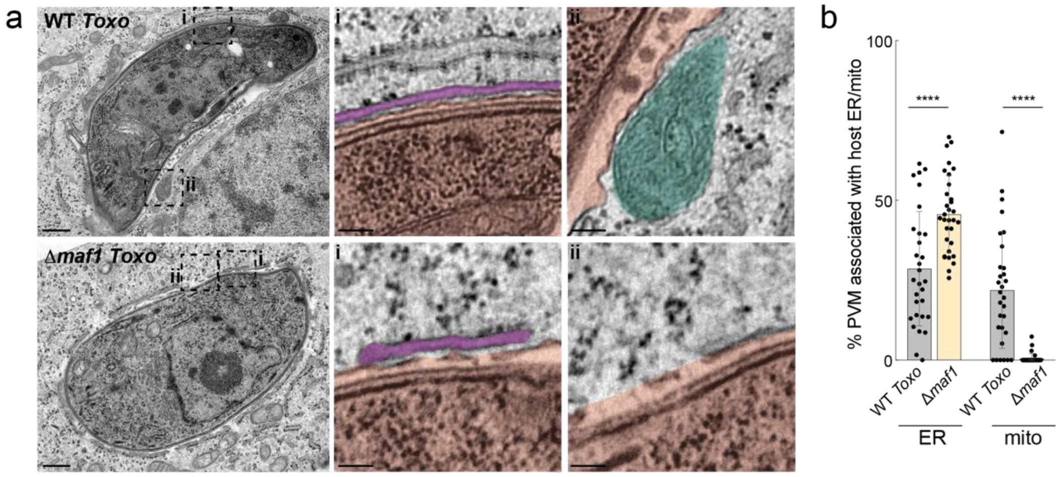

Supplementary Fig. 2: Δ*maf1* parasites have increased MCS with host ER

a, Representative electron micrograph images of ES-2 cells infected with *Toxo*$^{PVM\beta11}$ and Δ*maf1 Toxoplasma* at 4 hours post infection. MCS sites between the *Toxoplasma* parasite vacuole membrane (PVM) and (**i**) host ER and (**ii**) host mito. Scale bars: 500 nm; inset, 100 nm. Red, PV; purple, ER; turquoise, mito. **b,** Percentage of *Toxoplasma* PVM associated with host ER and mitochondria in images as in (**a**). EM data are mean ± SD from n=1 biological replicate (WT: 30; Δ*maf1*: 32 *Toxoplasma* vacuoles).. ****p< 0.0001 by means of unpaired two-tailed t-test.

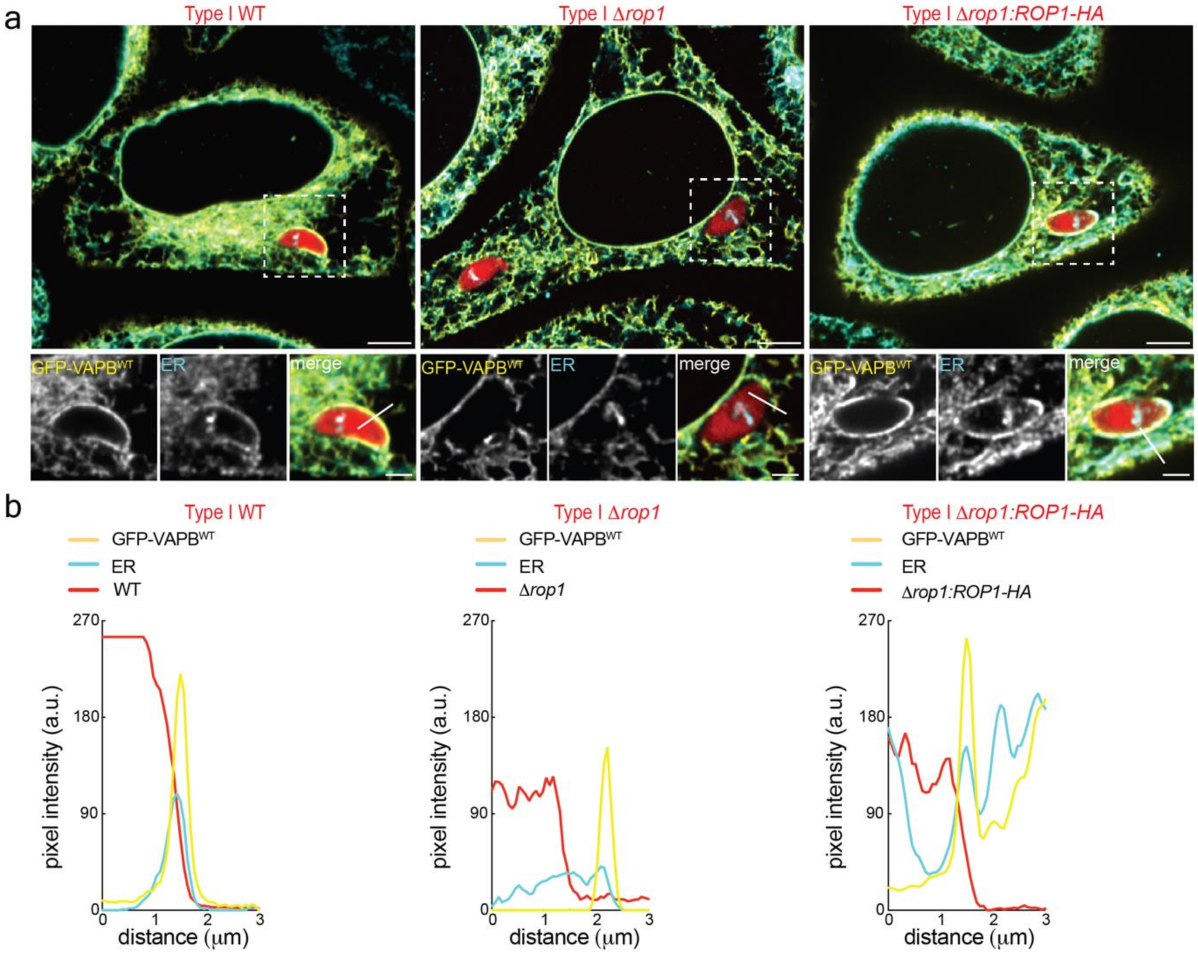

Supplementary Fig. 3: VAPB enrichment around Type I parasite vacuoles requires TgROP1

a, Representative immunofluorescence images of VAP DKO HeLa cells expressing GFP-VAPB^{WT} infected with *Toxoplasma* Type I WT (*Toxo*^{mCherry}), Δ*rop1*, Δ*rop1:ROP1-HA* parasites at 3 hours after infection. ER (calnexin). Scale bars, 5 μm; inset, 2 μm. **b,** Corresponding pixel intensity plots for white line in the (**a**) inset. Data is representative of n=1 biological replicate.

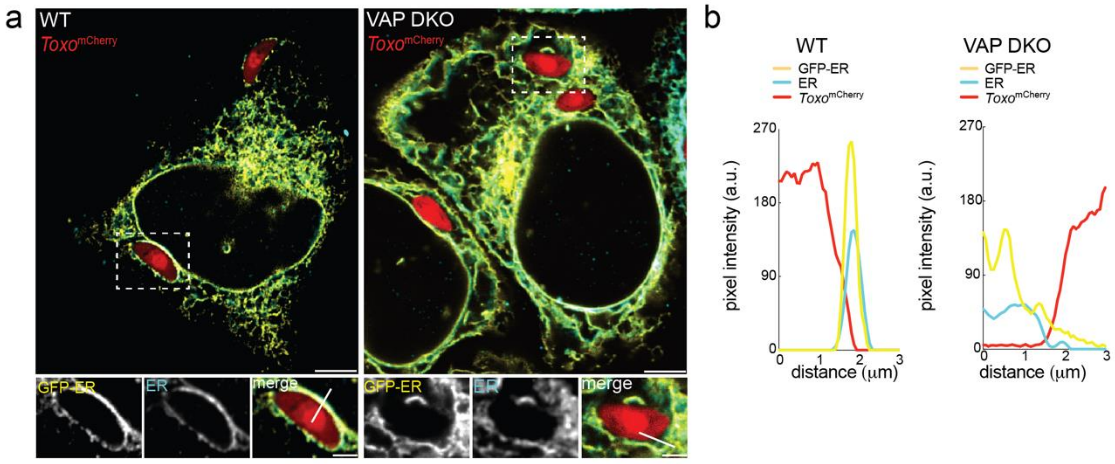

Supplementary Fig 4: Host ER enrichment around *Toxoplasma* is VAP-dependent

a, Immunofluorescence images of WT and VAP DKO HeLa cells expressing GFP on their ER membranes and infected with *Toxo*^{mCherry} at 3 hours post infection. ER (calnexin). Scale bars: 5 μm; inset, 2 μm. **b,** Corresponding pixel intensity plots for white line in the (**a**) inset. Data is representative of n=1 biological replicate.

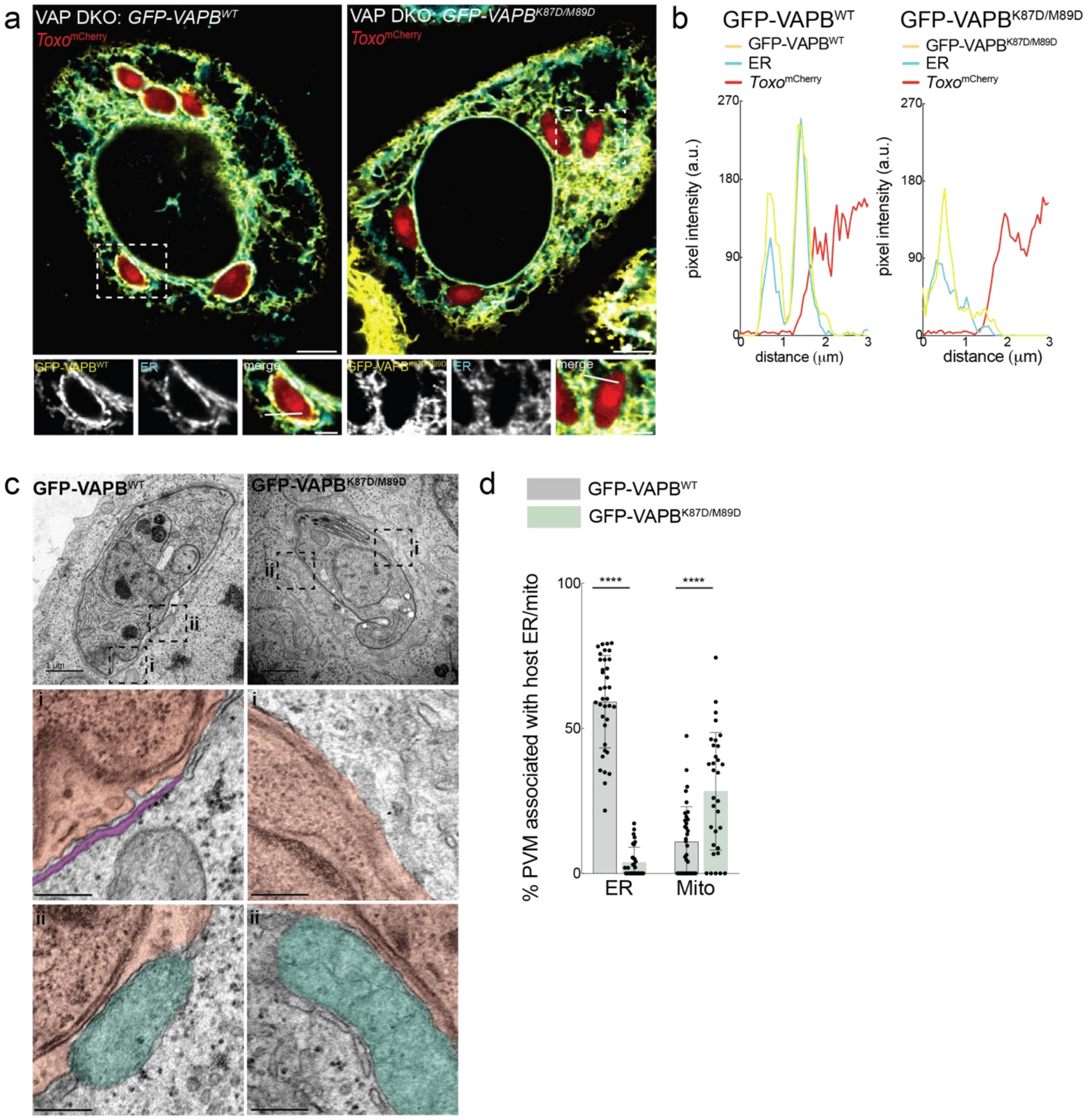

Supplementary Fig. 5: The VAPB MSP domain is critical for host ER-*Toxoplasma* MCS
a, Representative immunofluorescence images of GFP-VAPB^{WT} and GFP-VAPB$^{K87D/M89D}$ cells infected with *Toxo*^{mCherry} at 3 hours post infection (hpi). Data is representative of n=2 biological replicates. ER (calnexin). Scale bars: 5 µm; inset, 2 µm. **b,** Corresponding pixel intensity plots for white line in (**a**) inset. **c,** Representative electron microscopy images of GFP-VAPB^{WT} and GFP-VAPB$^{K87D/M89D}$ cells infected with *Toxo*^{mCherry} at 3 hpi. Scale bars: 1 µm; inset, 250 nm. Membrane contact sites between the *Toxoplasma* parasite vacuole membrane (PVM) and (**i**) host ER and (**ii**) host mito. Red, PV; purple, ER; turquoise, mito. **d,** Percentage of *Toxoplasma* PVM associated with host ER and mitochondria in images as in (**c**) from n=1 biological replicate (VAPB^{WT}: 36; GFP-VAPB$^{K87D/M89D}$: 32 *Toxoplasma* vacuoles). ****$p<0.0001$ by means of two-tailed unpaired t-test.

Additional Supplemenatry files included:

Supplementary Tables 1
List of gene targets and protospacer sequences used in the CRISPR screens.

Supplementary Table 2
List of *Toxoplasma* genes from the host mitochondria-*Toxoplasma* effector CRISPR screen showing the median log_2 fold change (log_2FC) in the sgRNA abundances between the GFP^{hi} and GFP^{neg} populations and robust ranking aggregation (RRA) scores.

Supplementary Table 3
List of *Toxoplasma* genes from the host ER-*Toxoplasma* effector CRISPR screen showing the median log_2 fold change (log_2FC) in the sgRNA abundance between the GFP^{hi} and GFP^{neg} populations and robust ranking aggregation (RRA) scores.

Supplementary Table 4
Analysis of transmembrane domain containing *Toxoplasma* rhoptry genes from the ER-*Toxoplasma* CRISPR screen for putative canonical or modified FFAT motifs, AlphaFold model scores and motif pLDDT values.

Supplementary Table 5
Proteomic analysis of GFP-IPs from uninfected and *Toxoplasma*-infected GFP-$VAPA^{WT}$-expressing VAP DKO HeLa cells.

Supplementary Table 6
Sequences of primers used in the study.

Supplementary Video 1
Time-lapse images of a live human foreskin fibroblast cell labelled with MitoTracker Deep Red and expressing OMM^{GFP-10} was infected with a parasite expressing $PVM^{\beta 11}$ ($Toxo^{PVM\beta 11}$). GFP is detected at the host mitochondria-*Toxoplasma* interface. Images were acquired every 3 minutes using a spinning-disk confocal microscope. PVM: parasite vacuole membrane; OMM: outer mitochondrial membrane. Scale bar, 5 µm

Supplementary Video 2
Time-lapse images of a live human foreskin fibroblast cell expressing GFP-$VAPA^{WT}$ and infected with Type I *Toxoplasma* parasites. Images were acquired every 6 min using a spinning-disk confocal microscope. Scale bar, 5 µm.
