# Supplementary material for: Toxoplasma effector TgROP1 establishes membrane contact sites with the endoplasmic reticulum during infection
Source: Nat Microbiol. 2025 Nov 25;10(12):3331–45. doi: 10.1038/s41564-025-02193-3 (PMC12669048; doi:10.1038/s41564-025-02193-3)
Supplement: Supplementary file 2 — Reporting Summary [file 41564_2025_2193_MOESM2_ESM.pdf]

nature portfolio

Corresponding author(s): Lena Pernas

Last updated by author(s): Aug 21, 2025

Reporting Summary

Nature Portfolio wishes to improve the reproducibility of the work that we publish. This form provides structure for consistency and transparency in reporting. For further information on Nature Portfolio policies, see our Editorial Policies and the Editorial Policy Checklist.

Statistics

For all statistical analyses, confirm that the following items are present in the figure legend, table legend, main text, or Methods section.

n/a	Confirmed	
☐	☒	The exact sample size (n) for each experimental group/condition, given as a discrete number and unit of measurement
☐	☒	A statement on whether measurements were taken from distinct samples or whether the same sample was measured repeatedly
☐	☒	The statistical test(s) used AND whether they are one- or two-sided<br>*Only common tests should be described solely by name; describe more complex techniques in the Methods section.*
☒	☐	A description of all covariates tested
☒	☐	A description of any assumptions or corrections, such as tests of normality and adjustment for multiple comparisons
☒	☐	A full description of the statistical parameters including central tendency (e.g. means) or other basic estimates (e.g. regression coefficient) AND variation (e.g. standard deviation) or associated estimates of uncertainty (e.g. confidence intervals)
☒	☐	For null hypothesis testing, the test statistic (e.g. F, t, r) with confidence intervals, effect sizes, degrees of freedom and P value noted<br>*Give P values as exact values whenever suitable.*
☒	☐	For Bayesian analysis, information on the choice of priors and Markov chain Monte Carlo settings
☒	☐	For hierarchical and complex designs, identification of the appropriate level for tests and full reporting of outcomes
☒	☐	Estimates of effect sizes (e.g. Cohen's d, Pearson's r), indicating how they were calculated

Our web collection on statistics for biologists contains articles on many of the points above.

Software and code

Policy information about availability of computer code

Data collection	Data are available in supplementary tables.
Data analysis	A description of analytical methods provided either in the main manuscript or in materials and methods.

For manuscripts utilizing custom algorithms or software that are central to the research but not yet described in published literature, software must be made available to editors and reviewers. We strongly encourage code deposition in a community repository (e.g. GitHub). See the Nature Portfolio guidelines for submitting code & software for further information.

Data

Policy information about availability of data

All manuscripts must include a data availability statement. This statement should provide the following information, where applicable:

- Accession codes, unique identifiers, or web links for publicly available datasets
- A description of any restrictions on data availability
- For clinical datasets or third party data, please ensure that the statement adheres to our policy

Data are available in supplementary tables.

Research involving human participants, their data, or biological material

Policy information about studies with human participants or human data. See also policy information about sex, gender (identity/presentation), and sexual orientation and race, ethnicity and racism.

Reporting on sex and gender	n/a
Reporting on race, ethnicity, or other socially relevant groupings	n/a
Population characteristics	n/a
Recruitment	n/a
Ethics oversight	n/a

Note that full information on the approval of the study protocol must also be provided in the manuscript.

Field-specific reporting

Please select the one below that is the best fit for your research. If you are not sure, read the appropriate sections before making your selection.

☒ Life sciences ☐ Behavioural & social sciences ☐ Ecological, evolutionary & environmental sciences

For a reference copy of the document with all sections, see nature.com/documents/nr-reporting-summary-flat.pdf

Life sciences study design

All studies must disclose on these points even when the disclosure is negative.

Sample size	Sample size determination was based on prior studies (i.e. for FACS analyses).
Data exclusions	No exclusions.
Replication	Replicates indicated.
Randomization	n/a
Blinding	n/a

Reporting for specific materials, systems and methods

We require information from authors about some types of materials, experimental systems and methods used in many studies. Here, indicate whether each material, system or method listed is relevant to your study. If you are not sure if a list item applies to your research, read the appropriate section before selecting a response.

Materials & experimental systems

n/a	Involved in the study	
☐	☒	Antibodies
☐	☒	Eukaryotic cell lines
☒	☐	Palaeontology and archaeology
☒	☐	Animals and other organisms
☒	☐	Clinical data
☒	☐	Dual use research of concern
☒	☐	Plants

Methods

n/a	Involved in the study	
☒	☐	ChIP-seq
☐	☒	Flow cytometry
☒	☐	MRI-based neuroimaging

Antibodies

Antibodies used	For Immunofluorescence analyses: Primary Abs: Calnexin (GeneTex: GTX109669 [C3], C-term) or Calnexin (Proteintech:10427-2-AP); VAPA (Proteintech15275-1-AP); TOMM70 (HPA:048020); VAPB (Proteintech: 14477-1-AP); HA (CST #3724, Roche 3F10); c-Myc (CST: 5605S, D84C12); Antisera of TgMAF1 15; HA (CST:3724, Roche 3F10); GFP (Takara Bio: 632380); TgROP1 (Abnova: MAB17504); TgROP6 (mouse monoclonal) were used at 1:300 or 1:2000 O/N. Secondary Abs: Alexa Fluor Plus 405, Alexa Fluor Plus 488, Alexa

Fluor Plus 594, Alexa Fluo Plus 647 (Thermo Fisher). For Immunoblotting: HMGCR (Sigma AMAB90619), TOMM70 (HPA048020); HA-HRP (Roche 12013819001); VAPA (Proteintech:15275-1-AP); VAPB (Proteintech: 14477-1-AP); Calnexin (Proteintech: 10427-2-AP); TgROP1(Abnova: MAB17504); TgROP6 (mouse monoclonal) and TgGra45 (Dr. D Soldati; U. of Geneva).

Validation

Validations were either indicated on relevant vendor website or validated in house (i.e. using knockout lines).

Eukaryotic cell lines

Policy information about cell lines and Sex and Gender in Research

Cell line source(s)

HeLa adenocarcinoma cells, ES-2 ovary clear cell carcinoma and human foreskin fibroblasts (HFFs) cells were obtained from ATCC (CCL-2, CRL-1978, and SCRC-1041, respectively); VAP A/B double knockout (VAP DKO) cells were a kind gift from Dr. Pietro Di Camelli. All cells were cultured at 37°C and 5% CO2 in Dulbecco's Modified Eagle's GlutaMAXTM medium and supplemented with 10% heat-inactivated FBS (Gibco: A3840402) and 100 U/ml Penicillin-Streptomycin (Thermo Fisher Scientific: 15070063) (referred to as cDMEM). ROP1 knockout parasites from Moritz Treeck (reference in manuscript), Type III parasites were a gift from Martin Blume.

Authentication

VAPA/B DKOs and ROP1 / ROP6 KO parasite lines were validated using PCR and immunoblot / immunofluorescence analysis.

Mycoplasma contamination

Cells were routinely tested for Mycoplasma infection by polymerase chain reaction (PCR) every two weeks.

Commonly misidentified lines
(See ICLAC register)

n/a

Plants

Seed stocks

Report on the source of all seed stocks or other plant material used. If applicable, state the seed stock centre and catalogue number. If plant specimens were collected from the field, describe the collection location, date and sampling procedures.

Novel plant genotypes

Describe the methods by which all novel plant genotypes were produced. This includes those generated by transgenic approaches, gene editing, chemical/radiation-based mutagenesis and hybridization. For transgenic lines, describe the transformation method, the number of independent lines analyzed and the generation upon which experiments were performed. For gene-edited lines, describe the editor used, the endogenous sequence targeted for editing, the targeting guide RNA sequence (if applicable) and how the editor was applied.

Authentication

Describe any authentication procedures for each seed stock used or novel genotype generated. Describe any experiments used to assess the effect of a mutation and, where applicable, how potential secondary effects (e.g. second site T-DNA insertions, mosiacism, off-target gene editing) were examined.

Flow Cytometry

Plots

Confirm that:

☒ The axis labels state the marker and fluorochrome used (e.g. CD4-FITC).

☒ The axis scales are clearly visible. Include numbers along axes only for bottom left plot of group (a 'group' is an analysis of identical markers).

☐ All plots are contour plots with outliers or pseudocolor plots.

☐ A numerical value for number of cells or percentage (with statistics) is provided.

Methodology

Sample preparation

For CRISPR screen: To perform the screen with technical duplicates, two vials of the split-GFP screen parasites (each considered as a technical duplicate) were thawed onto two T175 flasks of HFF monolayers. The next day the media of the flasks were changed to 25 µg/ml Mycophenolic acid and 50 µg/ml Xanthine (Sigma-Aldrich). Two days following treatment with selection media, the parasites were expanded by passing 2E6 parasites (to ensure a 1000x representation of guides) onto 6 T175 flasks of HFF monolayers. The next day, 300E6 OMM GFP1-10 ES-2 cells and ERM GFP1-10 ES-2 cells were plated in 15-cm dishes (8E6 cells/ dish). The next morning, split GFP-parasites from each technical replicate were used to infect 150E6 cells of each cell type at a low multiplicity of infection of 0.5 and left for 24 hours after infection. The following day, cells from each technical replicate (150 million cells) were trypsinized with accutase (to avoid clumpling) and pooled together into 50 ml falcons. The cells were fixed in 2% PFA for 5 minutes in FACS buffer with 5% accutase, spun down at 300 x g for 5 minutes to get rid of fixative. The cells were distributed into FACS tubes for sorting. The host mitochondria-Toxoplasma MCS screen cells were sorted using a BD FACSAria III sorter and the host ER-Toxoplasma MCS screen cells were sorted using a BD FACSFusion sorter. Gates were drawn to first sort for infected cells (mCherry fluorescence) and then all cells negative for GFP expression (GFPneg) and the top 20% of the GFP positive [GFP-high (GFPhi)] populations were sorted over the course of four days for both screens. Cell pellets were stored at -80°C; for growth analysis: For split GFP assays, monolayers of infected-ES2 or HeLa cells were rinsed with PBS, trypsinized and fixed in 2% paraformaldehyde in 3% FBS in 1XPBS (FACS buffer) for 5 min. After a spin at 300 rcf for 5 min, cells were resuspended in FACS buffer and 10,000 events were analyzed on a FACSFortessa using BD FACSDiva software for mCherry intensity and then tested for GFP expression. To assess parasite proliferation, monolayers of ES-2 cells infected with ToxomCherry parasites were left to grow for 24 hours post infection and harvested as

	previously described18. 10,000 events were analyzed on a FACSFortessa and the mCherry median fluorescence intensity (mFI) using BD FACSDiva software.
Instrument	BD FACSAria III; BD FACSFusion; FACS Fortessa
Software	BD FACSDiva software
Cell population abundance	Gates were drawn to first sort for infected cells (mCherry fluorescence) and then all cells negative for GFP expression (GFPneg) and the top 20% of the GFP positive [GFP-high (GFPhi)] populations were sorted over the course of four days for both screens; purity was assessed by rerunning populations and assessing GFP expression
Gating strategy	Cells were initially gated using forward scatter (FSC) versus side scatter (SSC) to exclude debris. The resulting population was then analyzed based on mCherry intensity to distinguish between uninfected (mCherry-; UI) and infected (mCherry+; INF) cells. Subsequently, both the UI and infected INF were assessed for their GFP expression levels; GFP-negative samples were used to draw gates for GFP-positive cells.

☒ Tick this box to confirm that a figure exemplifying the gating strategy is provided in the Supplementary Information.
