# Supplementary figures and images for: Toxoplasma effector TgROP1 establishes membrane contact sites with the endoplasmic reticulum during infection
Source: Nat Microbiol. 2025 Nov 25;10(12):3331–45. doi: 10.1038/s41564-025-02193-3 (PMC12669048; doi:10.1038/s41564-025-02193-3)

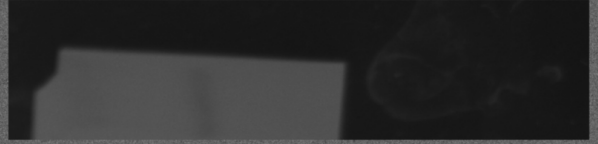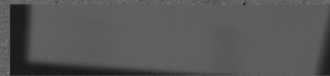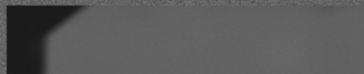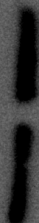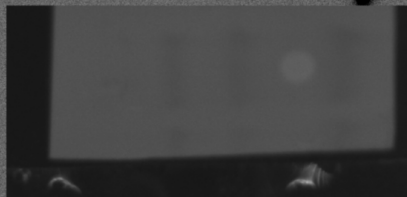

Supplement: Supplementary file 16 — Unprocessed blots. [file 41564_2025_2193_MOESM16_ESM.zip › SourceData_Fig4_unprocesedblots/Calnexin+Marker.pdf]

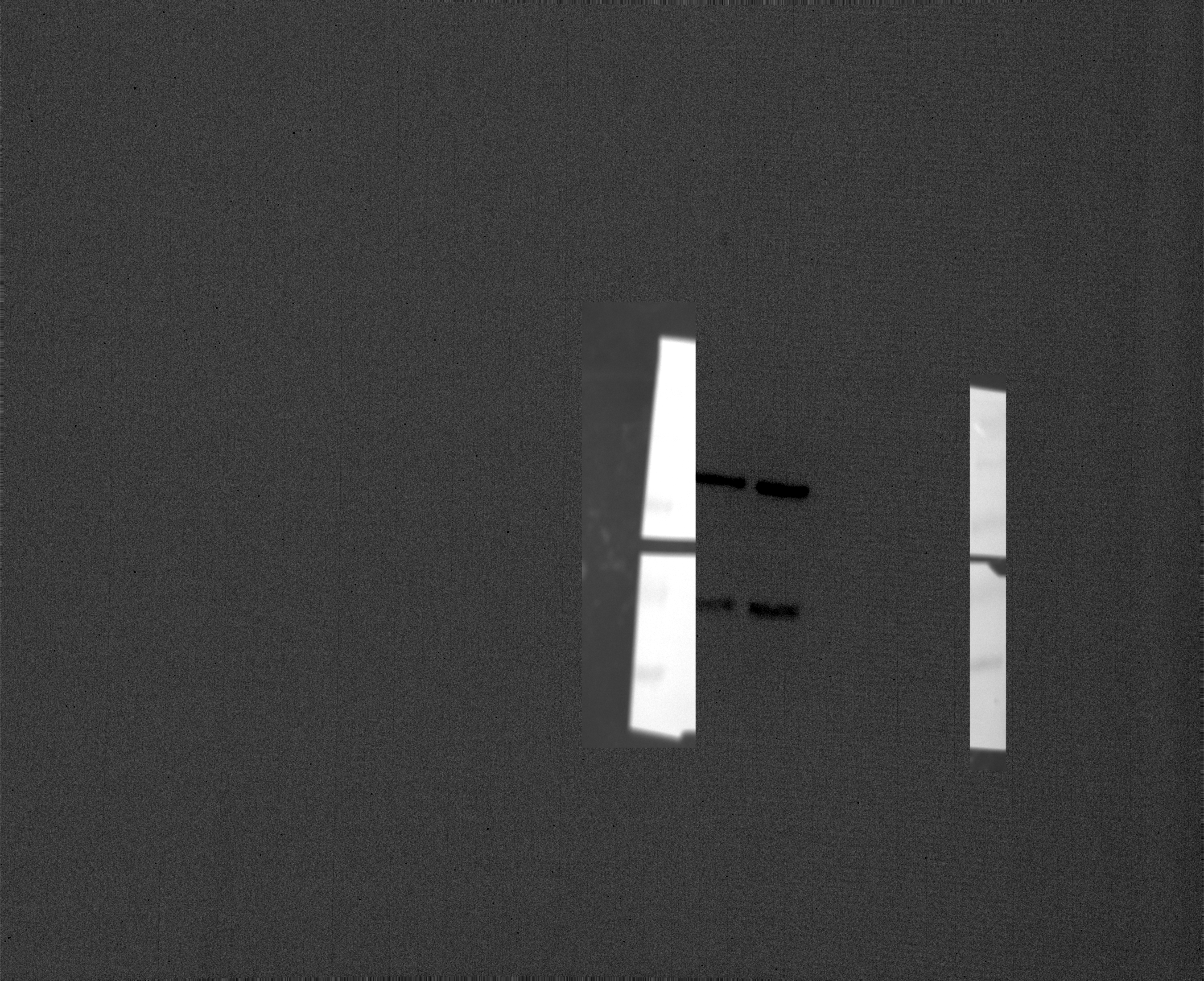

Supplement: Supplementary file 16 — Unprocessed blots. [file 41564_2025_2193_MOESM16_ESM.zip › SourceData_Fig4_unprocesedblots/HMGCR+TOM70+Marker.tif]

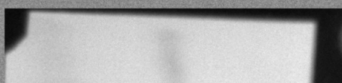

11  
11

Supplement: Supplementary file 16 — Unprocessed blots. [file 41564_2025_2193_MOESM16_ESM.zip › SourceData_Fig4_unprocesedblots/TgMAF1+Marker.pdf]

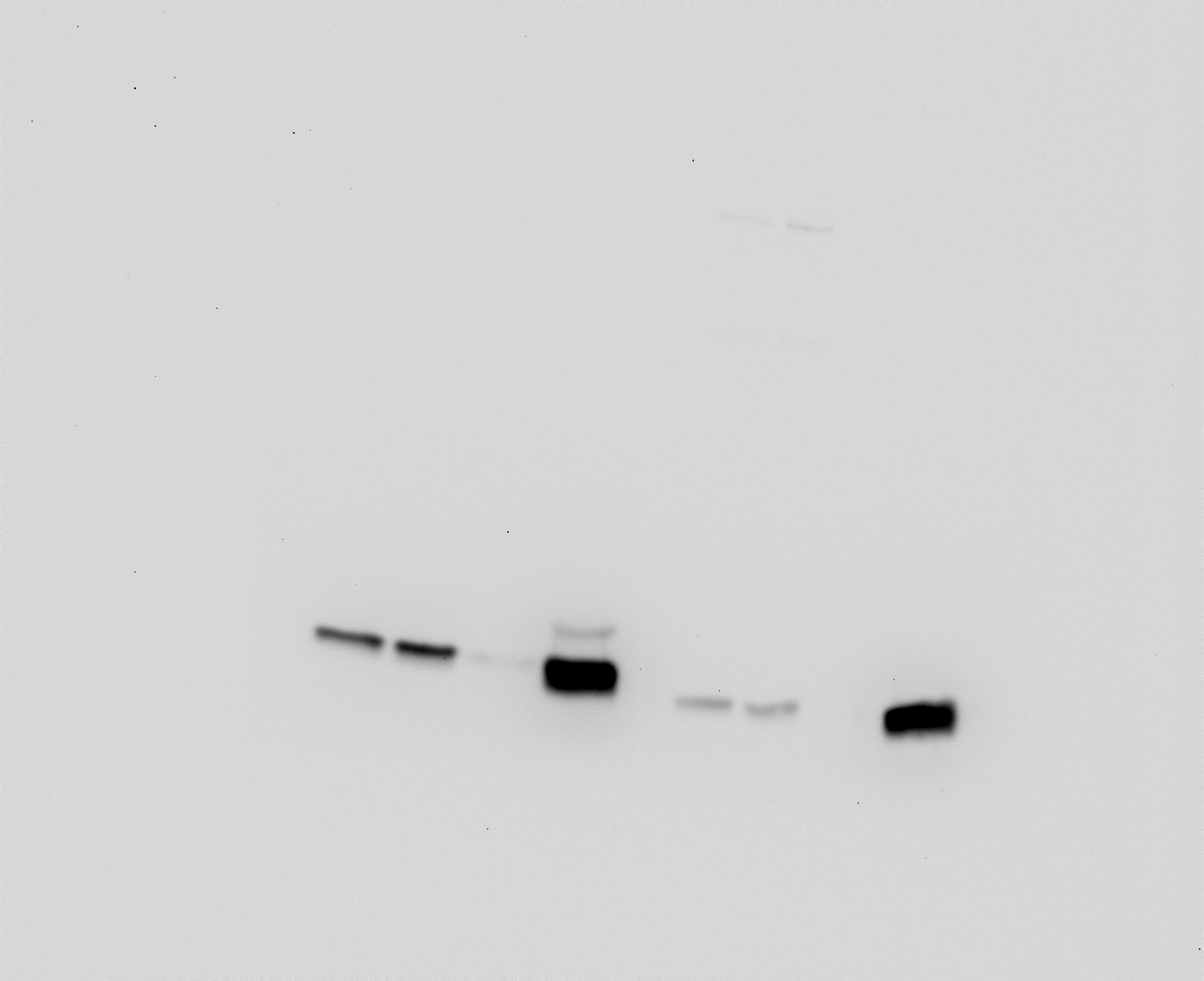

Supplement: Supplementary file 16 — Unprocessed blots. [file 41564_2025_2193_MOESM16_ESM.zip › SourceData_Fig4_unprocesedblots/VAPB+VAPA.tif]

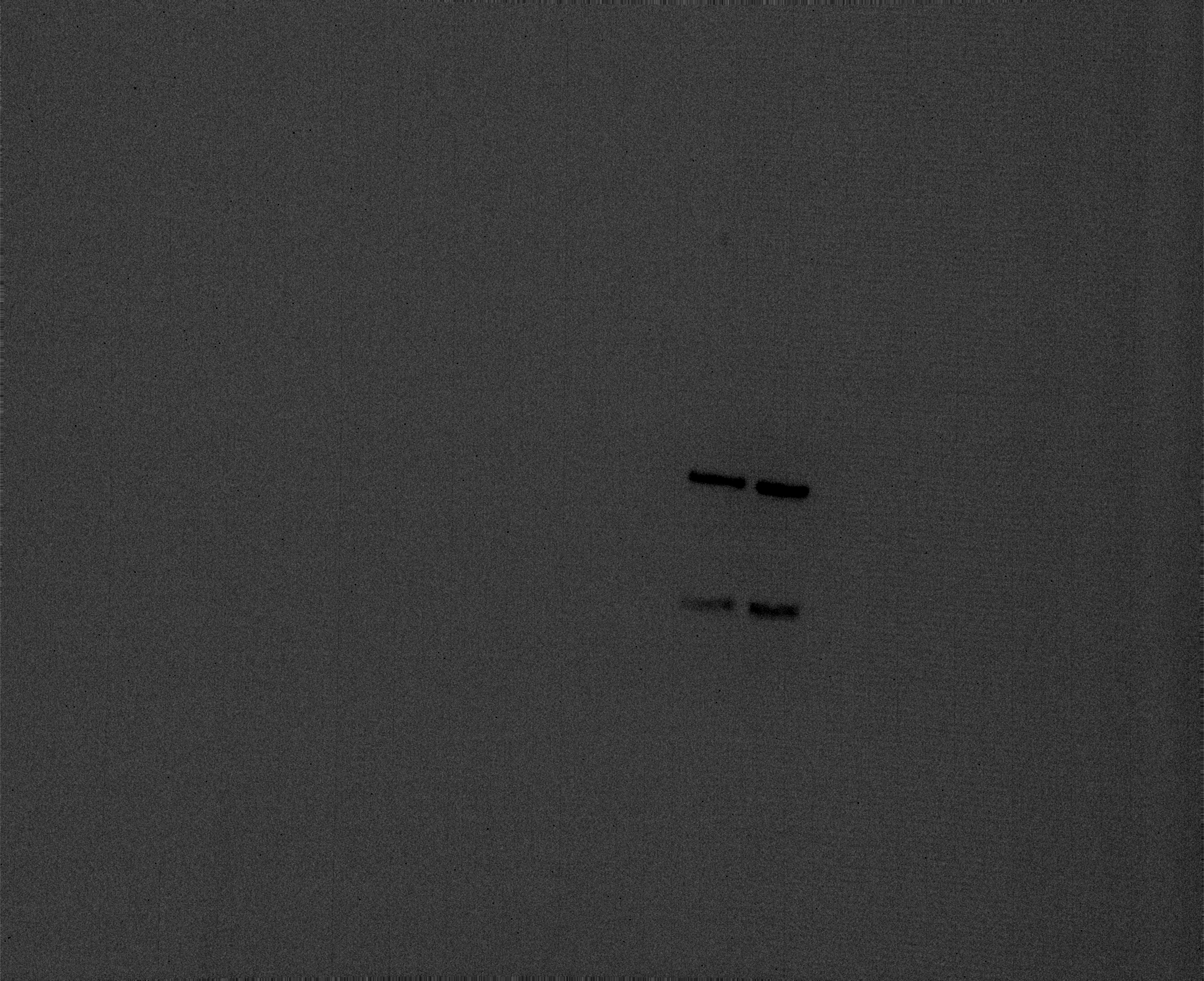

Supplement: Supplementary file 16 — Unprocessed blots. [file 41564_2025_2193_MOESM16_ESM.zip › SourceData_Fig4_unprocesedblots/HMGCR+TOM70.tif]

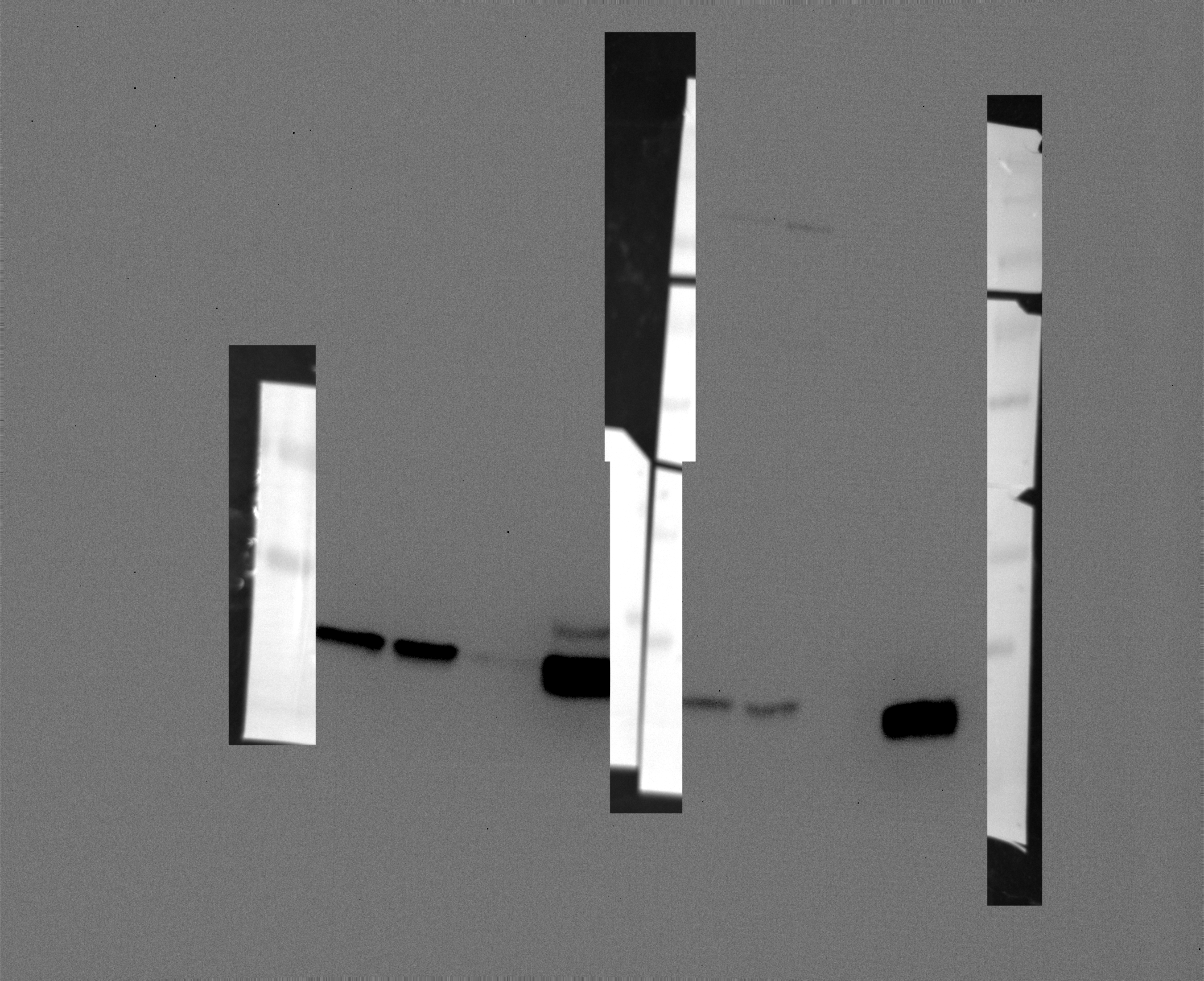

Supplement: Supplementary file 16 — Unprocessed blots. [file 41564_2025_2193_MOESM16_ESM.zip › SourceData_Fig4_unprocesedblots/VAPB+VAPA+Marker.tif]

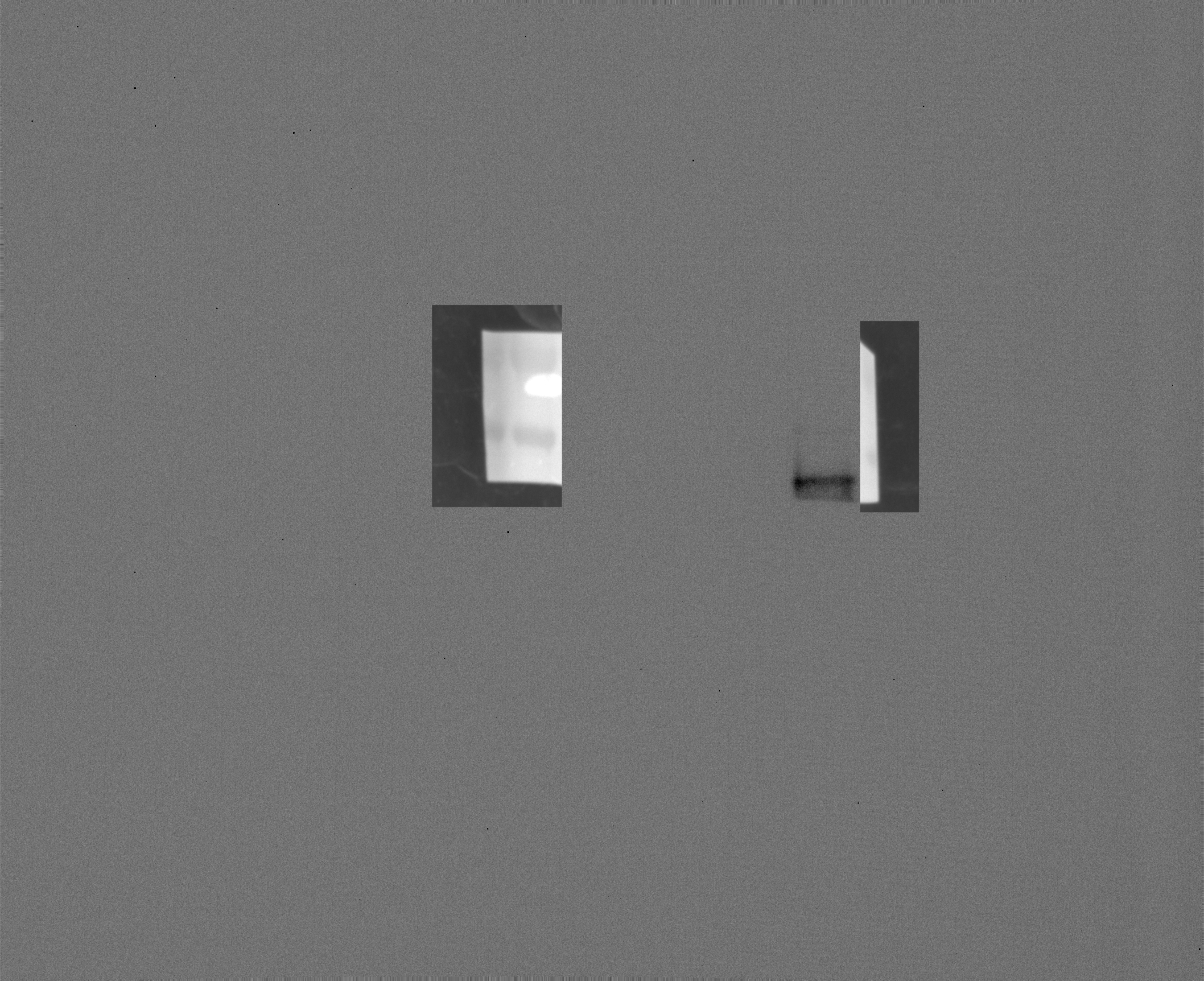

Supplement: Supplementary file 16 — Unprocessed blots. [file 41564_2025_2193_MOESM16_ESM.zip › SourceData_Fig4_unprocesedblots/HA_ROP1+Marker.tif]
